# Factors influencing the quality of life among women with cancer in Vietnam

**DOI:** 10.3389/fpsyg.2024.1431522

**Published:** 2024-12-20

**Authors:** Huyen Thi Hoa Nguyen, Tinh Thi Thanh Giap, Tran Ngoc Tran, Anh Chau Nguyen, Trung Quang Truong, Linh Khanh Bui, Duc Tran Quang, Debra J. Anderson

**Affiliations:** ^1^College of Health Sciences, VinUniversity (VinUni), Hanoi, Vietnam; ^2^Faculty of Health, University of Technology Sydney, Sydney, NSW, Australia; ^3^Faculty of Nursing and Midwifery, Hanoi Medical University, Hanoi, Vietnam; ^4^Faculty of Technology, Dong Nai Technology University, Bien Hoa, Vietnam

**Keywords:** quality of life, women’s health, cancer care, cross-sectional study, Vietnam

## Abstract

**Background:**

Cancer and its treatments significantly affect the quality of life (QoL) of patients. This remains understudied among Vietnamese women with cancer.

**Objectives:**

This study explored the QoL of Vietnamese women with cancer and identified key influencing factors.

**Method:**

This study analyzed 214 validated responses of women with cancer from hospitals in Viet Nam, assessing pain levels (Visualized Pain Scale), functional capacity (Karnofsky Performance Status Scale), and QoL (SF12). Analyses used SPSS 26.0, including *t*-tests, ANOVA, and hierarchical linear regression models.

**Results:**

SF12-PCS and SF12-MCS scores were 46.61 ± 9.70 and 46.96 ± 9.06. Pain score (*β* = −0.304, *p* < 0.001) and symptoms number (*β* = −0.311, *p* < 0.001) were key predictors of physical health. For mental health, functional status (*β* = 0.259, *p* < 0.001) and symptoms number (*β* = −0.311, *p* < 0.001) were significant. PCS was negatively correlated with age (*r* = −0.165, *p* = 0.016), number of symptoms (*r* = −0.220, *p* = 0.001), and pain (*r* = −0.444, *p* < 0.001). Mental health (MCS) was negatively correlated with the length of cancer diagnosis (*r* = −0.156, *p* = 0.036) and the number of symptoms (*r* = −0.362, *p* < 0.001). Both PCS and MCS positively correlated with functional status (*r* = 0.222, *p* = 0.001) and (*r* = 0.281, *p* < 0.001), respectively.

**Conclusion:**

Culturally sensitive care, pain management, and tailored support programs addressing physical, psychological, spiritual, and social aspects can enhance QoL for these women.

## 1 Introduction

### 1.1 Cancer’s impact on women worldwide

Cancer is a complex and multifaceted disease that poses significant challenges to individuals worldwide. Among those affected, women face distinct obstacles due to specific types of cancer that primarily afflict this population, including breast, cervical, ovarian, colorectal, and uterine malignancies (Torre et al., 2017). The global cancer incidence has reached around 18.1 million persons by 2020, with 8.8 million cases confirmed in women (World Cancer Research Fund International, 2020). In Southeast Asia alone, the International Agency for Research on Cancer reported that more than 1 million women are diagnosed with cancer per year, including breast, cervical, uterine, ovarian, colorectal, and lung cancers being the most frequently (World Cancer Research Fund International, 2020). In Vietnam, more than 80,000 women were diagnosed with new cases of cancer, comprising 50% of all newly reported cancer cases each year; particularly, breast, lung, and colorectum cancers were the top 3 most frequent cancers in Vietnamese accounting for 25.8, 9.1 and 9.0% of 83,647 newly diagnosed cases in 2020, respectively (Oncology IAFRO, 2021; Rivera-Franco and Leon-Rodriguez, 2018; Salama et al., 2019; Teo et al., 2018; Shah et al., 2019). Cancer prevalence in Vietnam reflects the global trend, underscoring the critical need to address the issues encountered by cancer patients in the country.

### 1.2 Challenges faced by women with cancer

During cancer, physical symptoms that result from the patient’s condition and therapies include pain, tiredness, nausea, hair loss, or menopausal-like symptoms (van den Beuken-van Everdingen et al., 2016; Niedzwiedz et al., 2019; Pai et al., 2020). Particularly, physical symptoms, psychological anguish, surgical treatments, and social support were important determinants of overall well-being and the quality of life of cancer survivors (Villar et al., 2017; dos Santos et al., 2019). Furthermore, the psychological distress associated with cancer diagnosis and treatment, such as worry, despair, and fear of recurrence, has a massive impact on women’s mental health (van den Beuken-van Everdingen et al., 2016; Niedzwiedz et al., 2019; Villar et al., 2017). Anywhere from 8–24% of cancer patients are living with depression (Walker et al., 2021; Zhao et al., 2014). A cancer diagnosis is related to an increased incidence of common mental illnesses in persons with no prior psychiatric history, which may have a detrimental effect on cancer therapy and recovery, as well as the quality of life and survival (Niedzwiedz et al., 2019). Depression affects up to 20% of cancer patients, compared to 5% in the general population globally (Pitman et al., 2018). Furthermore, a study conducted at the Vietnam National Cancer Hospital found that the prevalence of psychological discomfort in cancer patients is approximately 60%, depression 46%, and anxiety 27% (Vu et al., 2023). Additionally, women with cancer may suffer from comorbid conditions, such as osteoporosis, cardiovascular diseases, or secondary cancers (Han et al., 2021; Bui et al., 2015). Cancer can also impair women’s social lives in addition to their physical and mental health issues. They may endure social isolation, strained relationships, and disease stigma (Niedzwiedz et al., 2019; van Roij et al., 2019; Nalbant et al., 2021).

### 1.3 Factors influencing cancer-related quality of life (QoL)

As QoL measuring can provide a comprehensive picture of an individual’s overall well-being, more research has been conducted on patients’ QoL and its assessment has been widely used as an adjunct measure in oncology (Haslam et al., 2020; Phillips and Wong, 2020; Velikova et al., 1999). This is particularly important in women with cancer, whose QoL is profoundly affected due to cancer progress and its treatments (Seib et al., 2022). As such, numerous studies have been conducted around the world and in Vietnam to identify the risk factors and potential remedies to enhance the quality of life (QoL) of cancer patients. Key predictors of cancer-related quality of life include age, sex, race or ethnicity, marital status, socioeconomic level, treatment techniques, and access to healthcare services, as identified by a study published in JNCI Cancer Spectrum (Han et al., 2021). Similarly, in Vietnam, a study in 2015 on cancer patients in a national hospital stated that the quality of life of cancer patients is closely related to their educational levels, cancer stages, diagnosis duration, and treatment methods (Bui et al., 2015). Notably, patients with limited financial means are more vulnerable to decreased quality of life, even after cancer treatment, as some risk factors may persist (Lathan et al., 2016). Consequently, cancer patients, particularly those with low income, generally experience lower health-related quality of life compared to non-cancer women, and even if they survive and recover from cancer, their quality of life remains lower than that of age-matched women in the general population (Ngan et al., 2022).

### 1.4 Research gap and significance of the study

Given the tripling number of new cases and deaths of cancer in Vietnam over the past 30 years, it is imperative to understand the quality of life of female cancer patients in this context (Pham et al., 2019). However, there remains a paucity of studies examining cancer-related quality of life among women in Vietnam. Vietnam’s socio-cultural context, healthcare infrastructure, and economic conditions are different from those in other Western and Asian countries where many QoL studies have been done (Huyen et al., 2021). Vietnamese women are faced with distinctive challenges including conventional gender roles, stigma related to cancer and lack of available supportive care services that could have a major influence on their quality of life (Nguyen, 2017; Phung et al., 2023; Nguyen et al., 2024). Therefore, the purpose of this study is to:

1. Describe the quality of life of women with cancer in Vietnam and
2. Identify the factors influencing their quality of life.

The findings from this study will contribute to the existing body of knowledge and provide valuable insights into the specific needs and concerns of Vietnamese women with cancer. By understanding these factors, healthcare practitioners and policymakers can design and implement targeted interventions to improve the overall quality of life and treatment outcomes for women with cancer in Vietnam.

## 2 Method

### 2.1 Research design

This study adopts a cross-sectional design to assess the quality of life (QoL) of women living with cancer in Vietnam. 214 validated responses were included for data analysis. The convenient sampling method was applied.

### 2.2 Participants

The participants of this study are eligible with inclusion criteria including (1) Vietnamese women over 18 years old and older, (2) who have been diagnosed with cancer including breast cancer, gynecological cancer, and hematological cancer, (3) who have finished at least one phase of intensive cancer treatment in the last 6 months, and (4) who are willing to participate in the study. The study excluded those who are diagnosed with mental illness.

### 2.3 Time and location

Data collection was conducted from September to December 2023 in person at several hospitals in Hanoi, Vietnam, which have an oncology department.

### 2.4 Instruments

General information includes sociodemographic data, medical and family history related to cancer, the number of symptoms after cancer, and the frequency of these symptoms were collected.

Participants’ pain levels were evaluated using the Visualized Pain Scale, a 10-point scale ranging from 0 to 10, with higher scores indicating higher pain levels. This is a widely used tool for assessing pain levels and has been used in various studies and clinical settings (Paschali et al., 2020; Kataria et al., 2024).

The Karnofsky Performance Status Scale was used to measure the level of functional capacity in women living with cancer (Schag et al., 1984). This scale rates the level of functional capacity on a scale from 20 to 100%, with higher percentages indicating better functional performance status.

The Short Form 12 (SF-12), a 12-item short-form derived from the original Short Form-36 survey, was used to measure participants’ individual perceptions of their quality of life (Turner-Bowker and Hogue, 2014). The SF-12 tool assesses aspects of both physical and mental health. This instrument demonstrated high internal consistency (Cronbach’s alpha = 0.81) in previous study (Nejati et al., 2021).

### 2.5 Data collection

The researchers obtained a list of women with cancer who had received treatment at the selected hospitals, along with their contact information (usually phone numbers). Eligible participants were invited to participate in the study through phone calls or in-person invitations at the hospital. Participants who agreed to participate and met the inclusion criteria were provided with an information sheet about the study and a consent form. After obtaining written consent, participants completed the survey questionnaire in the presence of the researchers. The data collection process took approximately 15 min per participant.

### 2.6 Data analysis

Data analyses were conducted using IBM SPSS version 26.0. In two-sided tests, a statistical significance cutoff of 0.05 was employed. Characteristics and health-related variables of participants were described by presenting either the number (percentage) or the mean ± standard deviation values. The assumption of normality was verified using the Shapiro–Wilk test before conducting parametric tests (all *p*-values >0.05). To examine the differences in physical health (PCS) and mental health (MCS) scores among subgroups with varying participant characteristics and health-related variables, independent t-tests and one-way ANOVA were utilized. Additionally, the Tukey HSD test was performed as a post-hoc analysis. Pearson’s correlation coefficients were computed to determine the strength of the linear association between participants’ continuous variables (such as age, length of cancer diagnosis, and pain score) and QoL (PCS and MCS scores). Furthermore, hierarchical linear regression models were computed to assess the relationships between QoL (PCS and MCS scores) and potential predictors. The residuals of the regression models were examined to ensure they met the Gauss-Markov conditions. The analysis was conducted in two blocks. The first block consisted of participant characteristic variables, while the second block included additional participant health-related variables.

### 2.7 Ethical approval

The Institutional Ethical Review Board for BioMedical Research of Vinmec International General Hospital-VinUniversity approved this study (No.75/2022/QD-VMEC, dated 26^th^ July 2022).

## 3 Results

### 3.1 Descriptive statistics of study participants

Table 1 presents the characteristics of 214 participants. The average age of the participants was approximately 49.63 ± 10.84 years, with a relatively even distribution between urban (56.5%) and rural (43.5%) regions. The majority of the participants identified as no religious (88%), and over half of them held a bachelor’s degree or higher. Approximately 87% were currently employed, with 45% engaged in skilled labor and 42% involved in unskilled labor. Regarding monthly income, it ranged from 0 to 100 million VND, with an average of 8.3 ± 10.77 million VND. Almost all participants had insurance coverage (98.6%) and had someone at home to provide support (79%).

**Table 1:** Characteristics and medical history of participants (*n* = 214).

Variable	Mean ± SD or *n* (%)
Characteristics of participants	
Age	49.63 ± 10.84 (range: 18–77)
Living area	
Urban	121 (56.5)
Rural	93 (43.5)
Religion	
No religion	188 (87.9)
Buddhism	17 (7.9)
Catholicism	8 (3.7)
Protestantism	1 (0.5)
Education	
Elementary	6 (2.8)
Secondary	42 (19.6)
High school	55 (25.7)
College and higher	111 (51.9)
Employment	
Unemployment	28 (13.1)
Unskilled labor	89 (41.6)
Skilled labor	97 (45.3)
Marital status	
Single	10 (4.7)
Divorced/windowed	17 (7.9)
Married/living with a partner	187 (87.4)
Personal income/month (million VND)	8.30 ± 10.77 (range: 0 to 100)
Insurance (yes)	211 (98.6)
Family member support at home^&^	
None	45 (21.0)
Husband	107 (50.0)
Parents-in-law	16 (7.5)
Parents	10 (4.7)
Caregivers	6 (2.8)
Others	56 (26.2)
Medical history of participants	
Length of cancer diagnosis (year)	3.02 ± 3.22 (Range 0 to 22)
BMI	21.94 ± 2.56 (Range 15.4 to 34.8)
Having chronic disease(s) (not cancer)	49 (22.9)
Type of cancer^&^	
Cervical cancer	6 (2.8)
Breast cancer	180 (84.1)
Others	15 (7.0)
Cancer stage	
Stage 1	35 (24.8)
Stage 2	79 (56.0)
Stage 3	21 (9.8)
Stage 4	6 (2.8)
Current therapy^&^	
Surgery	90 (42.1)
Chemotherapy	146 (68.2)
Radiation therapy	52 (24.3)
Immunotherapy	5 (2.3)
Hormone therapy	32 (15.0)
Others	36 (16.8)
Smoking (yes)	2 (0.9)
Drinking (yes)	2 (0.9)
Exercise (yes)	171 (79.9)
Family member diagnosed with cancer	58 (27.1)
Strategy used to monitor and manage symptoms^a^	
None	10 (4.7)
Periodical health check	201 (93.9)
Technological device	11 (5.1)
Internet-based support	6 (2.8)
Sleeping pattern in last week	
Time to fall asleep (minute)	29.21 ± 20.04 (Range: 0 to 120) Mode: 30 (*n* = 68, 39.8%)
Length of sleeping at night (hour)	6.82 ± 1.25 (Range: 2 to 11) Mode: 7.0 (*n* = 65, 31.6%)
No. of time wake up in the midnight	1.65 ± 1.52 (Range: 0 to 12)

^aMultiple responses.^

Additionally, the medical history of the participants is presented. On average, the duration of cancer diagnosis was 3.02 ± 3.22 years. The majority of participants (84%) had been diagnosed with breast cancer. Among the four cancer stages, the second stage was the most reported (56%). As for the current cancer treatment methods, chemotherapy and surgery were the most frequently employed options, with utilization rates of 68 and 42%, respectively. In terms of lifestyle, a significant proportion of participants reported not smoking or drinking (99%), and 80% engaged in regular exercise. The average duration of nightly sleep was 6.8 ± 1.25 h. Alongside their cancer diagnosis, 23% of the participants also experienced other chronic illnesses.

Table 2 shows the results of the survey on eight commonly experienced symptoms in cancer patients. The study found that hair loss, fatigue, and sleep disturbances were prevalent issues among the participants. In terms of the frequency of symptoms (counted for symptoms that appeared at least once a week – 2 point, to always – 6 point), the average number of symptoms reported was 4.97 ± 2.55, with a maximum possible score of 8. The average pain score was 1.23 ± 1.74, indicating that 44% of participants experienced pain to some degree, categorized as mild, moderate, or severe in 31, 12, and 1% of cases, respectively. The average score for functional status was 88%, suggesting a relatively high level of functional capacity. However, 24% of participants rated their functional capacity at 70% or lower (70%: unable to carry on normal activity or to do active work, to 20%: very sick, hospital admission necessary, active supportive treatment necessary). In terms of quality of life, the average score for physical health (PCS) was 46.31 ± 9.70, while the score for mental health (MCS) was 46.96 ± 9.06.

**Table 2:** Health conditions of participants (*n* = 214).

Symptoms after treatment		Mean ± SD or *n* (%)
Fatigue		2.76 ± 1.37
Loss of appetite		2.42 ± 1.42
Pain		2.25 ± 1.39
Sleep disturbances		2.69 ± 1.53
Hair loss		3.03 ± 1.91
Nausea and/or vomiting		1.98 ± 1.35
Sexual health issues		1.87 ± 1.20
Mood disturbances		1.97 ± 1.30
Number of symptoms (6-point Likert scale: 1 – never to 6 – always, Items counted if scored > 1)		4.97 ± 2.55
Pain (scale: 0 to 10)		1.23 ± 1.74 (Range: 0 to 8)
Level	No (0)	120 (56.1)
	Mild (1–3)	67 (31.3)
	Moderate (4–6)	25 (11.7)
	Severe (7–10)	2 (0.9)
Functional status (scale 0 to 100%)		88.32 ± 12.37 (Range: 40 to 100)
Level	F1 (80–100%)	188 (87.9)
	F2 (50 to 70%)	24 (11.2)
	F3 (20 to 40%)	2 (0.9)
Quality of life (SF12)		
Physical component summary score (PCS)		46.31 ± 9.70
Mental health component summary score (MCS)		46.96 ± 9.06

### 3.2 Physical health and mental health in subgroups of variables

In terms of physical health, the PCS score exhibited significant differences among subgroups of six variables: religion, marital status, BMI, chronic disease, pain, and functional status. For mental health (MCS) scores, significant differences were revealed in subgroups categorized by the length of cancer diagnosis and functional status, as shown in Table 3.

**Table 3:** Quality of life according to subgroups of variables (*n* = 214).

Variable	*n*	PCS		MCS	
		Mean ± SD	*p*	Mean ± SD	*p*
Age					
< 40^a^	42	47.28 ± 9.62	0.131	45.13 ± 9.29	0.132
40-49^b^	74	46.82 ± 9.27		47.88 ± 8.25	
50-59^c^	52	47.51 ± 9.93		45.23 ± 9.27	
60-69^d^	38	43.95 ± 9.84		48.83 ± 9.27	
≤70^e^	8	39.98 ± 9.92		50.25 ± 10.94	
Living area					
Urban	121	46.06 ± 10.30	0.668	47.45 ± 9.01	0.357
Rural	93	46.64 ± 8.90		46.30 ± 9.14	
Religion					
No	188	47.14 ± 9.30	0.010***	46.74 ± 9.14	0.877
Yes	26	40.34 ± 10.61		48.52 ± 9.06	
Education					
High school and lower	103	45.98 ± 9.93	0.626	46.69 ± 8.50	0.686
College and higher	111	46.62 ± 9.51		47.19 ± 9.58	
Employment					
Unemployment^a^	28	43.36 ± 1.16	0.153	50.23 ± 9.8	0.103
Unskilled labor^b^	89	47.41 ± 9.33		46.5 ± 8.48	
Skilled labor^c^	97	46.15 ± 9.81		46.83 ± 9.44	
Marital status					
Single^a^	10	44.29 ± 11.22	0.017* b ≠ c**	47.10 ± 8.83	0.791
Divorced/windowed^b^	17	40.18 ± 9.99		48.39 ± 10.05	
Married/living with a partner^c^	187	46.98 ± 9.43		46.82 ± 9.19	
Personal income					
≤ 8 million VND	85	44.64 ± 8.75	0.749	49.11 ± 8.65	0.947
> 8 million VND	43	44.06 ± 11.06		49.14 ± 8.79	
Family member support at home					
None^a^	44	47.40 ± 8.93	Multiple responses	45.60 ± 7.99	Multiple responses
Husband^b^	107	44.37 ± 9.01		48.95 ± 8.70	
Parents-in-law^c^	16	50.65 ± 7.80		46.06 ± 10.28	
Parents^d^	10	44.47 ± 9.36		51.15 ± 8.14	
Caregivers^e^	6	43.81 ± 14.49		47.23 ± 14.56	
Others^f^	56	48.90 ± 10.56		44.93 ± 9.86	
Length of cancer diagnosis					
< 3 years	125	47.09 ± 9.04	0.834	48.25 ± 9.19	0.004
≥ 3 years	56	46.77 ± 11.21		44.10 ± 8.31	
BMI					
≤ 18.4^a^	11	40.42 ± 10.39	0.030* a ≠ b**	43.57 ± 8.92	0.442
18.5–24.9^b^	179	47.04 ± 9.41		47.11 ± 9.09	
≥ 25.0^c^	24	43.57 ± 10.52		47.37 ± 8.99	
Other chronic disease					
Yes	49	43.40 ± 8.99	0.016***	48.76 ± 8.43	0.114
No	165	47.18 ± 9.76		46.43 ± 9.19	
Cancer stage					
Stage 1^a^	35	44.67 ± 9.47	0.178	48.88 ± 9.32	0.360
Stage 2^b^	79	43.07 ± 9.35		48.75 ± 8.30	
Stage 3^c^	21	46.27 ± 8.11		49.61 ± 7.41	
Stage 4^d^	6	37.61 ± 9.86		42.79 ± 6.79	
Current therapy					
Surgery^a^	90	47.23 ± 9.31	Multiple responses	44.95 ± 8.33	Multiple responses
Chemotherapy^b^	146	46.98 ± 9.35		45.60 ± 8.75	
Radiation therapy^c^	52	47.78 ± 9.03		44.62 ± 7.40	
Immunotherapy^d^	5	45.35 ± 7.89		41.36 ± 9.96	
Hormone therapy^e^	32	42.66 ± 11.27		51.55 ± 7.40	
Others^f^	36	43.24 ± 8.99		51.20 ± 8.25	
Exercise					
Yes	171	46.61 ± 9.39	0.136	47.03 ± 9.10	0.805
No	43	44.34 ± 10.74		46.65 ± 9.02	
Family member diagnosed with cancer					
Yes	58	44.94 ± 11.01	0.208	46.66 ± 9.88	0.775
No	156	46.82 ± 9.14		47.06 ± 8.76	
Strategy used to monitor and manage symptoms					
None^a^	10	38.23 ± 10.17	Multiple responses	44.07 ± 8.15	Multiple responses
Periodical health check^b^	201	46.78 ± 9.53		47.17 ± 9.07	
Technological device^c^	11	40.21 ± 8.79		50.53 ± 9.07	
Internet-based support^d^	6	44.16 ± 7.28		45.27 ± 8.69	
Pain					
No^a^	120	49.68 ± 8.73	<0.001* a ≠ b ≠ c**	46.38 ± 9.70	0.511
Mild^b^	67	44.77 ± 7.82		47.98 ± 8.57	
Moderate and severe^c^	27	35.19 ± 8.94		46.97 ± 10.71	
Functional status					
F1	188	47.32 ± 9.10	<0.001***	47.42 ± 8.93	0.045***
F2 and F3	26	39.05 ± 10.94		43.63 ± 9.51	

*One-way ANOVA. **Post-hoc analysis (Tukey HSD test). ***t-test. ^a–f^refers to the categories of each variable to show the significant level at 0.05.

### 3.3 Correlation between QoL and variables

Table 4 shows the significant associations between PCS score and several variables. Specifically, PCS demonstrated a negative correlation with age (*r* = −0.165, *p* = 0.016), number of symptoms (*r* = −0.220, *p* = 0.001), pain (*r* = −0.444, *p* < 0.001), and a positive correlation with functional status (*r* = 0.222, *p* = 0.001). Furthermore, the MCS score displayed significant correlations with three variables. These included a negative correlation with the length of cancer diagnosis (*r* = −0.156, *p* = 0.036), number of symptoms (*r* = −0.362, *p* < 0.001), and a positive correlation with functional status (*r* = 0.281, *p* < 0.001).

**Table 4:** Correlation matrix of QoL.

Variable	*r* (*p*)	
	PCS	MCS
Age	−0.165 (0.016)	0.115 (0.095)
Personal income	−0.040 (0.653)	0.002 (0.978)
Length of cancer diagnosis	−0.014 (0.851)	−0.156 (0.036)
Length of sleeping at night	0.063 (0.365)	−0.125 (0.074)
Number of symptoms	−0.220 (0.001)	−0.362 (<0.001)
Pain	−0.444 (<0.001)	0.034 (0.625)
Functional status	0.222 (0.001)	0.281 (<0.001)

### 3.4 Predictors of QoL

Table 5 presents the predictors of the physical health score (PCS) and mental health score (MCS). In block 1, four characteristic variables were included as independent variables. The model accounted for 4.2% of the variance in PCS scores, and only religion was found to be a significant predictor. Moving to block 2, seven variables pertaining to the participants’ health conditions were added. This expanded model explained 29.1% of the variance in PCS scores. Two significant predictors were identified: the pain score (*β* = −0.304, *p* < 0.001) and the number of symptoms (*β* = −0.311, *p* < 0.001).

**Table 5:** Factors influencing PCS and MCS (*n* = 214).

	Predictor	Block 1			Block 2		
		*B*	*SE*	*β*	*B*	*SE*	*β*
PCS	Age	−0.097	0.079	−0.107	−0.128	0.072	−0.141
	Religion (following a religion = 1)	−4.506	2.247	−0.170*	−2.267	2.356	−0.075
	Employment (unemployment = 1)	−1.061	2.625	−0.035	−3.069	1.989	−0.116
	Marital status (married/living with a partner = 1)	2.864	2.216	0.108	2.536	2.030	0.095
	BMI (normal = 1)				3.374	1.805	0.139
	Chronic disease (yes = 1)				−0.253	1.638	−0.012
	Stage of cancer				0.124	0.979	0.010
	Physical exercise (yes = 1)				2.172	1.700	0.095
	Pain score				**−1.552**	**0.385**	−0.304***
	Functional status				0.050	0.056	0.070
	No. of symptoms				**−1.213**	**0.290**	−0.311***
	*R^2^* (adjusted)	0.042			0.291		
	*R^2^* change				0.249		
	F for change in *R^2^*	2.523*			6.212***		
MCS	Age	0.090	0.072	0.100	0.079	0.071	0.089
	Employment (unemployment = 1)	4.532	2.435	0.149	2.074	2.272	0.068
	Length of cancer diagnosis				−0.278	0.200	−0.099
	Chronic disease (yes = 1)				2.619	1.599	0.116
	Functional status				0.205	0.056	0.259***
	Length of sleeping at night				−0.002	0.506	−0.0003
	No. of symptoms				−1.101	0.256	−0.311***
	*R^2^* (adjusted)	0.031			0.218		
	*R^2^* change				0.187		
	F for change in *R^2^*	3.772*			7.833***		

*p < 0.05, ***p < 0.001. Bold values represent the standardized regression coefficients or beta coefficients.

Regarding mental health, the model accounted for 21.8% of the variance in the MCS score, and two significant predictors were identified. These predictors include functional status (*β* = 0.259, *p* < 0.001) and the number of symptoms (*β* = −0.311, *p* < 0.001).

## 4 Discussion

The main goal of this study was to investigate the QoL among Vietnamese women living with cancer and explore the factors influencing their QoL. The research aims to fill a gap in the understanding of QoL in this specific population, providing valuable insights for healthcare practitioners and policymakers to improve the well-being of these women.

The study found relatively high mean scores for both physical health (mean of PCS is 46.31) and mental health (mean of MCS is 46.96), indicating that these women reported a generally favorable QoL. The findings align with some existing studies in Vietnam and worldwide, which also reported relatively high QoL scores in certain cancer populations. However, differences with other findings highlight the importance of considering individual, cultural, and regional factors when assessing QoL. For instance, a study conducted in Vietnam utilizing the same SF-12 instrument on a predominantly female population of type 2 diabetes mellitus patients found that the presence of at least one diabetic complication correlated with diminished scores across various domains of SF-12, particularly in the aspect of MCS (Pham et al., 2020). Another study from India reveals that cancer patients, particularly those from disadvantaged populations, experience poor health-related QoL outcomes (Sharma and Purkayastha, 2017). It is indeed possible that financial distress and belonging to minority populations in India could add to the burdens of cancer patients. The demographic characteristics of the participants in our study were diverse, but they might not have experienced the same level of financial distress or minority representation as observed in the Indian study.

PCS demonstrated a negative correlation with age, number of symptoms, and pain, and a positive correlation with functional status. This indicates that older age, a higher number of symptoms, and greater pain were associated with poorer physical QoL. Similarity, the MCS score displayed a negative correlation with the length of cancer diagnosis, number of symptoms, and a positive correlation with functional status, suggesting that women who have been diagnosed with cancer for a longer period and experienced more symptoms tend to have lower MCS scores. This could indicate that over time, cancer survivors may find it more challenging to maintain the mental strategies they initially employed to cope with their diagnosis, higher symptom burden and related challenges, while better functional status was associated with better physical QoL and mental health.

The significant predictors identified in the regression analysis for PCS and MCS provide valuable insights into the determinants of QoL in this population. Notably, religion, pain score, number of symptoms, and functional status emerged as significant factors impacting QoL outcomes. The influence of religion on QoL suggests the potential role of spiritual and cultural beliefs in coping with strategies (Kumar et al., 2023; Yeom et al., 2022; Fradelos et al., 2021; Majda et al., 2022; Moysés et al., 2023). As cancer and its treatment can lead to changes in women’s lifestyles and mental health, healthcare practitioners should be sensitive to the spiritual and cultural backgrounds of patients to provide comprehensive and patient-centered care (Nguyen et al., 2024). Also, the impact of pain score, number of symptoms, and functional status on QoL indicates that interventions targeting pain management, symptom control, and rehabilitation programs may significantly enhance the QoL of women with cancer. These findings align with previous research on factors influencing QoL in cancer patients, emphasizing the universal importance of symptom management and functional well-being in shaping QoL outcomes in this population (Tao et al., 2016; Wang et al., 2022; Stout et al., 2021; Odynets et al., 2019). Interestingly, our findings revealed no association between exercise and quality of life, despite substantial evidence suggesting that regular exercise significantly enhances quality of life among women with cancer (Hoa Nguyen et al., 2024). This discrepancy may be due to the exercise variable in our study not being assessed with a validated instrument, which could introduce bias in the results. Future studies should consider using validated, culturally appropriate tools to measure exercise in this population for more accurate insights.

The demographic characteristics of the participants provide insightful context for understanding the study findings. A noteworthy aspect is the substantial proportion of participants who identified as no religious (88%). This suggests that the majority of the study population might employ coping strategies other than religion, such as seeking secular forms of emotional support or drawing strength from family, social networks, personal beliefs, and their innate resilience. The relatively diverse urban–rural distribution, educational backgrounds, employment status, and income levels reflect the heterogeneity of the sample, which contributes to the generalizability of the results. The broad representation of participants allows for a comprehensive understanding of QoL experiences in different socioeconomic and cultural contexts. Tailored strategies can be developed to address the specific needs of different subgroups within the population, considering their unique demographic characteristics and life circumstances.

### 4.1 Clinical implications

Religion, pain, number of symptoms, and functional status are revealed as significant factors of QoL among cancer with women in Vietnam; therefore, healthcare practitioners should consider these elements when providing treatment to deliver holistic and patient-centered care. Moreover, programs and interventions should be sensitive to the spiritual and cultural backgrounds of patients, and focus on pain management, symptom control, and rehabilitation. The diverse demographic characteristics of the participants imply that specialized strategies should be tailored to address the individual requirements needs of different subgroups within the population, taking into account their distinct demographic traits and living situations. For example, cultural tailored care plans that incorporating spiritual care into treatment, giving access to spiritual advisors, or providing spaces for meditation for Buddhist patients. It is also crucial that healthcare providers be trained in cultural competency so as to understand and respect the Vietnamese women’s cultural norms and values. Connecting women with community resources, such as support groups and cancer community, can assist in navigating available services. Specifically, urban cancer patients may benefit from support groups, survivorship programs, and wellness centers within city limits. Meanwhile, rural patients may need telehealth services, transportation assistance, and community-based outreach.

### 4.2 Study limitations

The study’s sample size of 214 participants is relatively small, and a larger sample could provide more robust results. Additionally, there may be other factors not explored in the analysis, such as social support and coping mechanisms, which could also impact QoL outcomes and require further investigation. Nevertheless, the study’s findings offer valuable insights into the QoL of Vietnamese women with cancer and suggest areas for potential improvement in healthcare and support services to enhance their overall well-being.

## 5 Conclusion

This study sheds light on the quality of life (QoL) of Vietnamese women living with cancer and its implications. Culturally sensitive care, effective pain management, and comprehensive support programs can enhance well-being. Tailored interventions for diverse subgroups should be considered. Holistic approaches addressing physical, psychological, spiritual, and social aspects are vital. Collaboration between practitioners and policymakers can lead to patient-centered strategies, ultimately improving QoL for Vietnamese women with cancer.

## Data Availability

The datasets presented in this article are not readily available because the data contain sensitive and protected information that could compromise the privacy of the participants. Requests to access the datasets should be directed to the corresponding author.
