# Correction: Factors influencing the quality of life among women with cancer in Vietnam

**DOI:** 10.3389/fpsyg.2026.1988933

**Published:** 2026-09-30

**Authors:** Huyen Thi Hoa Nguyen, Tinh Thi Thanh Giap, Tran Ngoc Tran, Anh Chau Nguyen, Trung Quang Truong, Linh Khanh Bui, Duc Tran Quang, Debra J. Anderson

**Affiliations:** 1 College of Health Sciences, VinUniversity (VinUni), Hanoi, Vietnam; 2 Faculty of Health, University of Technology Sydney, Sydney, NSW, Australia; 3 Faculty of Nursing and Midwifery, Hanoi Medical University, Hanoi, Vietnam; 4 Faculty of Technology, Dong Nai Technology University, Bien Hoa, Vietnam

**Keywords:** quality of life, women's health, cancer care, cross-sectional study, Vietnam

In the **abstract**, the text stated:

“**Method:** In 2022, this study analyzed 214 Vietnamese women with cancer from four hospitals, assessing pain levels (Visualized Pain Scale), functional capacity (Karnofsky Performance Status Scale), and QoL (SF12). Analyses used SPSS 26.0, including t-tests, ANOVA, and hierarchical linear regression models.”

This has been corrected to read:

“**Method:** This study analyzed 214 validated responses of women with cancer from hospitals in Viet Nam, assessing pain levels (Visualized Pain Scale), functional capacity (Karnofsky Performance Status Scale), and QoL (SF12). Analyses used SPSS 26.0, including *t*-tests, ANOVA, and hierarchical linear regression models.”

The funder VinUniversity to Dr. Huyen Thi Hoa Nguyen was erroneously omitted and the **Funding** statement stated the following:

“The author(s) declare that no financial support was received for the research, authorship, and/or publication of this article.”

The correct **Funding** statement is as follows:

“The author(s) declared that financial support was received for this work and/or its publication. Financial support was provided by VinUniversity to HN. The funder had no role in the conceptualization, decision to publish, or the preparation of the manuscript.”

There was text erroneously missing from **section 2 Method, subsection 2.1 Research design, Paragraph 1:**

“This study adopts a cross-sectional design to assess the quality of life (QoL) of 214 women living with cancer in Vietnam. The convenient sampling method was applied.”

It has now been replaced to read:

“This study adopts a cross-sectional design to assess the quality of life (QoL) of women living with cancer in Vietnam. 214 validated responses were included for data analysis. The convenient sampling method was applied.”

There was text erroneously included in **section 2 Method, subsection 2.3 Time and location, Paragraph 1**

“Data collection was conducted from September to December 2022 in person at several hospitals in Hanoi, Vietnam, which have an oncology department. The hospitals included were National Oncology Hospital, Hanoi Medical University Hospital, Vinmec Times City International Hospital, and Hanoi Obstetrics and Gynecology Hospital.”

It has now been replaced to read:

“Data collection was conducted from September to December 2023 in person at several hospitals in Hanoi, Vietnam, which have an oncology department.”

There was text erroneously included in **section 3 Results, subsection 3.1 Descriptive statistics of study participants, Paragraph 1:**

“Out of the 284 questionnaires completed, 214 (75%) were analyzed due to minimal or no missing data. Table 1 presents the characteristics of these 214 participants. The average age of the participants was approximately 49.63 ± 10.84 years, with a relatively even distribution between urban (56.5%) and rural (43.5%) regions. The majority of the participants identified as no religious (88%), and over half of them held a bachelor's degree or higher. Approximately 87% were currently employed, with 45% engaged in skilled labor and 42% involved in unskilled labor. Regarding monthly income, it ranged from 0 to 100 million VND, with an average of 8.3 ± 10.77 million VND. Almost all participants had insurance coverage (98.6%) and had someone at home to provide support (79%).”

It has now been corrected to read:

“Table 1 presents the characteristics of 214 participants. The average age of the participants was approximately 49.63 ± 10.84 years, with a relatively even distribution between urban (56.5%) and rural (43.5%) regions. The majority of the participants identified as no religious (88%), and over half of them held a bachelor's degree or higher. Approximately 87% were currently employed, with 45% engaged in skilled labor and 42% involved in unskilled labor. Regarding monthly income, it ranged from 0 to 100 million VND, with an average of 8.3 ± 10.77 million VND. Almost all participants had insurance coverage (98.6%) and had someone at home to provide support (79%).”

The original version of this article has been updated.

